# Assessment of olfactory fluctuations in a clinical context

**DOI:** 10.1007/s00405-022-07462-z

**Published:** 2022-06-05

**Authors:** Anna Kristina Hernandez, Lena Juratli, Antje Haehner, Julien W. Hsieh, Basile N. Landis, Thomas Hummel

**Affiliations:** 1 Smell and Taste Clinic, Department of Otorhinolaryngology, Technische Universität Dresden, Haus 5, Fetscherstrasse 74, 01307 Dresden, Germany; 2 Rhinology-Olfactology Unit, Department of Otorhinolaryngology, University of Geneva, Geneva, Switzerland; 3 University of Michigan Medical School, Ann Arbor, MI USA

**Keywords:** Olfaction, Fluctuation, Smell, Sinonasal, Postviral

## Abstract

**Purpose:**

The aim of the study was to investigate whether olfactory fluctuations (OF) are pronounced in patients with sinonasal olfactory dysfunction (OD).

**Methods:**

The retrospective investigation included patients aged 18 years or older, who consulted a tertiary referral center for olfactory loss. Patients with normal smell function were excluded. Patients answered a structured questionnaire about their olfactory symptoms, with specific questions related to the presence of OF and its average frequency, amplitude, duration, time since most recent OF, and associated symptoms of self-reported OF. Patients also underwent clinical evaluation including a structured medical history and physical examination including nasal endoscopy. In addition, we assessed orthonasal olfactory function using Sniffin’ Sticks, and gustatory function using “taste sprays”.

**Results:**

Participants included 131 men and 205 women (*n* = 336), aged 18 to 86 years (mean 50, SD 16). Patient-reported fluctuations occurred most frequently in sinonasal (38%), idiopathic (29%), and postviral (29%) OD. Amplitude of OF was highest in postviral OD (*p* = 0.009). Average frequency, duration, and the time since the most recent fluctuation were not significantly different between groups (all *p*’s > 0.42). Odor discrimination (*p* = 0.002) and identification (*p* = 0.017) scores were higher among those individuals with OF.

**Conclusion:**

Amplitude of OF may help distinguish postviral from other causes of OD, especially in patients presenting with equivocal symptoms of sinonasal disease.

## Introduction

Olfactory dysfunction (OD) is defined as an impaired, distorted, and/or absent sense of smell. A subset of OD includes olfactory fluctuation (OF), which is characterized by the reversible transition between different states of olfactory function, ranging from present or decreased olfactory function to a total loss of function within a given period of time. OF has been implicated to be related to an increasing number of diseases, including allergic rhinitis [1, 2], Parkinson’s disease [3], Alzheimer’s disease [4], and multiple sclerosis [5, 6]. Still, OF is most strongly associated with inflammatory or obstructive sinonasal disease [1, 2, 7–11], and has been previously accepted as a pathognomonic sign of chronic rhinosinusitis (CRS) [1, 11].

Although OF is common among patients with OD, very little data-based analysis of OF has been done [10] and no current structured model of clinical workup for OF assessment exists. Due to the wide range of amplitude, duration, and frequency of symptoms, OF patients can be a clinical challenge. This challenge is aggravated by the lack of standardized questionnaires to diagnose OF and their potential cause. Hence, the aim of the present study was to investigate the presence of OF in patients with various causes of OD.

## Materials and methods

The retrospective study design was approved by the Institutional Review Board (IRB) of TU Dresden and was conducted according to the principles expressed in the Declaration of Helsinki.

### Participants

The study included adult patients of at least 18 years of age, who consulted at the Smell and Taste Clinic, Department of Otorhinolaryngology, TU Dresden for sinonasal, post-infectious, post-traumatic, and idiopathic olfactory dysfunction. Patients who only experienced isolated cases of transient fluctuations that were not related to persistent olfactory dysfunction (e.g., acute viral rhinitis), not requiring medical consultation were not included in the study.

### Questionnaire

Participants answered a structured questionnaire regarding their olfactory and gustatory symptoms, with specific questions related to the presence of OF and its average frequency, amplitude, duration, time since most recent OF, and associated symptoms of self-reported OF (Table 1). Cronbach’s alpha was 0.948.

**Table 1:** Olfactory fluctuation questionnaire

Screening	My smelling ability is not good, but is much better from time to time in the short-term	☐ Yes ☐ No (if “no”, the questionnaire ends here)
When yes		
Frequency	I experience changes in my sense of smell…?	☐ Daily ☐ Only once a week ☐ Only once a month ☐ Yearly
Amplitude	In comparison to my typical situation, the short-term improvement in smelling is…?	﻿☐ Only slightly better ﻿☐ Somewhat better ﻿☐ Much better
Duration	The short-term improvement in smelling lasts…?	﻿☐ Seconds ﻿☐ Minutes ﻿☐ Hours ﻿☐ Days
Time since most recent olfactory fluctuation	When was the last time you experienced such a fluctuation in smelling ability?	﻿☐ Hours ago ﻿☐ Days ago ﻿☐ Weeks ago ﻿☐ Months ago ﻿☐ Years ago
Associated symptoms of self-reported olfactory fluctuations	What are these odor fluctuations related to?	﻿☐ Physical exercise ﻿☐ Heat ﻿☐ Cold ﻿☐ Medication ﻿☐ Showering ﻿☐ Humidity ﻿☐ Spicy food ﻿☐ Other: __________

### Clinical evaluation

The clinical workup included a standardized, systematic medical history followed by a detailed ENT physical examination including nasal endoscopy [7]. Olfactory testing was done using the “Sniffin’ Sticks” [12] (Burghart Messtechnik, Holms, Germany), which is comprised of tests for odor threshold (T), discrimination (D), and identification (I). Patients also underwent screening for gustatory dysfunction using the “taste sprays” (sweet, sour, salty, bitter) [13, 14]. If deemed necessary, their cranial CT scans or MR images were examined.

### Data analysis

Statistical analyses were performed using SPSS ver. 28.0 (IBM SPSS Statistics for Windows, Vs. 28.0; IBM Corp., Armonk, NY, USA). Kruskal–Wallis tests and Mann–Whitney *U* test were done to determine which causes of OD had the most OFs, as well as to explore possible differences in amplitude, frequency, duration, and time since most recent OF. Finally, comparisons of TDI scores and taste scores with OF were performed using multivariate ANOVA, with an alpha level of 0.05 considered as statistically significant. We treated the following responses as “missing” values: patients who did not know or did not give an answer. Imaging data were collected irregularly for purposes of diagnosis but were not included in the analysis.

## Results

**Fig. 1:**
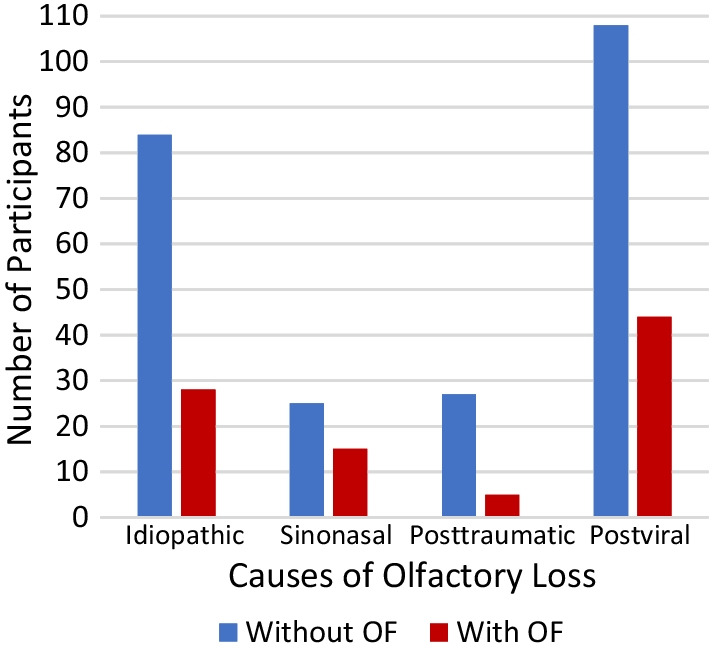
Frequency of patients with and without olfactory fluctuations (OF) among various causes of olfactory loss

The study participants included 131 men and 205 women (*n* = 336), whose ages ranged from 18 to 86 years (mean 50 years, SD 16). The causes of olfactory loss for the study sample included: sinonasal disease (*n* = 40), viral infections of the upper respiratory tract (*n* = 152), idiopathic dysfunction (*n* = 112), and head trauma (*n* = 32). Patient-reported olfactory fluctuations occurred most frequently in sinonasal (38%, *n* = 15), postviral (29%, *n* = 44), and idiopathic (25%, *n* = 28) OD, whereas they were observed less in patients with post-traumatic olfactory loss (16%, *n* = 5) (Fig. 1). The three groups where fluctuations were most frequently observed exhibited a significant difference in the amplitude of the fluctuations. This was highest in those with postviral OD (*H*(2) = 9.45, *p* = 0.009), with a mean rank of 41.1 for postviral, 23.9 for sinonasal, and 22.0 for idiopathic. The three groups did not differ with regard to other OF parameters such as frequency (*H*(2) = 1.01, *p* = 0.60), duration (*H*(2) = 0.30, *p* = 0.86), and the time since the most recent fluctuation (*H*(2) = 1.70, *p* = 0.43). The amplitude of OF was significantly higher in postviral compared to sinonasal OD (*U* = 113, *p* = 0.006).

**Fig. 2:**
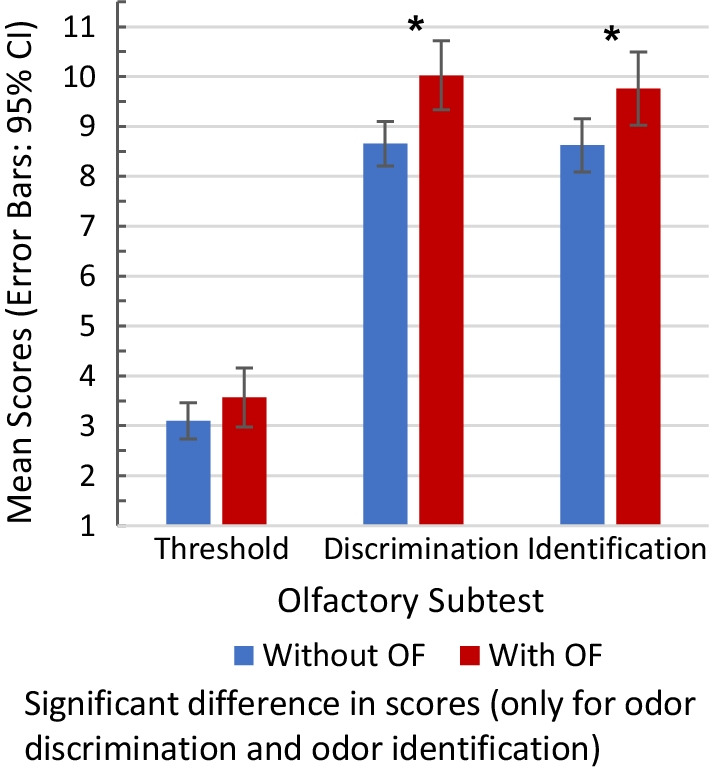
Sniffin’ Sticks scores in subjects with and without olfactory fluctuation (OF)

In comparison to patients without OF, patients with OF had better olfactory function for odor discrimination (*F*_1,311_ = 10.14, *p* = 0.002) and identification (*F*_1,311_ = 5.78, *p* = 0.017) scores, but not for odor threshold scores. Figure 2 presents descriptive plots of the discrimination and identification subtest scores. There was no significant difference in taste scores between those with and without OF (*F*_1,311_ = 0.645, *p* = 0.422).

## Discussion

Various hypotheses have been proposed to explain OF; however, the exact mechanism of OF remains unknown. The present study confirms previous findings by Hsieh et al. [10] that self-reported OF is strongly, but not exclusively, associated with sinonasal disorders. In addition to previous studies, our findings show that OF is most frequently observed in sinonasal OD, compared to postviral and idiopathic OD.

The pathophysiology of sinonasal OD involves an impairment of nasal airflow and inflammation of the olfactory neural pathway [10, 11, 15, 16]. Airflow to the olfactory region was found to increase when airway patency improved at critical areas, such as the olfactory cleft and the internal nasal valve [17]. However, airway patency does not seem to greatly influence olfactory dysfunction, except in cases of marked nasal obstruction [18]. This suggests that airflow is not the sole factor influencing olfaction, as is evident in cases when smell function rarely returns to normal levels in CRS patients after nasal surgery and corticosteroid treatments [19]. Inflammation and mucosal remodeling in the area of the olfactory neuroepithelium has also been shown to occur in CRS, negatively affecting olfactory function [15, 16, 20].

It has been proposed that certain activities (e.g., exercise, showering, sexual intercourse) or anti-inflammatory therapy (e.g., corticosteroids) and physiologic processes (e.g., nasal cycle, endogenous hormone secretion) eliciting nasal decongestion or affecting mucosal conditions in the nose can cause OF [1, 8, 9, 21]. This implies the presence of an intact olfactory neural pathway, from the receptors at the olfactory neuroepithelium up to the olfactory processing centers in the brain, which is a prerequisite for olfactory function [1, 11, 22]. We theorize that OF occurs in patients who experience poor olfactory function, in the setting of a combined effect of nasal obstructive disease and an underlying inflammatory condition, such that the damage to the olfactory mucosa is not too severe to be permanent. OFs indicate that olfactory function is a continuum that can shift from the state of normal function to reversible impairment, more frequently observed in some diseases than others. In our study, patients reporting OF were more likely to have better olfactory function versus those without OF, as expressed by the higher discrimination and identification subtest scores in those with OF. This emphasizes that for OF to occur, it requires the presence of baseline olfactory function. In this case, it is likely that healthy olfactory neuroepithelium is present and extensive metaplasia has not yet occurred [16], thereby making olfactory fluctuations possible.

Amplitude of OF may help distinguish postviral from sinonasal OD. Nasal polyps were found not to be associated with OF, but they appear to be a marker of more severe smell loss [1, 2]. Thus, OF amplitude might be particularly useful in differentiating postviral OD versus newly-diagnosed CRS without nasal polyps (CRSsNP). Postviral OD would be slightly less prone to OF [1, 11] but would have higher amplitude of fluctuations, presenting with more sudden olfactory loss after an upper respiratory tract infection, and would be more likely to present with parosmia/phantosmia [22, 23]. On the other hand, CRSsNP would likely present with moderate-amplitude fluctuations, gradually worsening olfactory loss, and would be less likely to present with parosmia/phantosmia [21].

Odor distortions are known to frequently occur after postviral olfactory loss [24]. We hypothesize that the reported increased amplitude of OF in postviral OD may be an indicator of a phase of recovery where possibly regenerating olfactory epithelium temporarily produces an increased amplitude of distorted odor perceptions which patients may perceive as periods of a more sensitive sense of smell or heightened smelling abilities.

Previous work by Apter et al. [2] evaluated subjective OF using a questionnaire, among 90 patients with allergic rhinitis only (*n* = 30) versus those with allergic rhinitis and olfactory dysfunction without sinonasal disease (*n* = 30), and those with allergic rhinitis and olfactory dysfunction with sinonasal disease (*n* = 30). Their findings suggested that the frequency of self-reported OF is positively correlated with sinonasal disease severity [2]. In contrast to this, we found no significant association between the frequency of self-reported OF and a causative underlying OD disease.

In sinonasal diseases, particularly chronic rhinosinusitis, OF may be due to diverse inflammatory processes driven primarily by dysfunctional interactions at the level of the sinonasal mucosa. Various host and environmental factors may influence these conditions that are conducive for the development of inflammation [25]. Inflammation not only occurs in the respiratory epithelium, but in the olfactory epithelium as well [15, 16]. The ability of the olfactory neuroepithelium to repair and regenerate, may account for some of the OF observed in sinonasal disease. Transient olfactory improvement has also been observed in CRS patients receiving corticosteroid treatment [1, 6, 22].

One of the challenges to investigating OF is the lack of a clear, universally accepted definition of the concept. To our knowledge, most of the articles cited in this study mention the word “fluctuation”, but only the study by Hsieh et al. [10] provided a definition for this concept. In general, fluctuation is defined as several changes in amount, size, quality etc. that happen frequently. However, one would also question whether this fluctuation is instantaneous and fleeting or happening over a period of days or weeks; whether it denotes a shift between the state of being able to smell well versus smelling less or not being able to smell at all; or if it happens as bursts of improved olfaction amidst a state of persistent poor olfaction. It is hard to be certain if all reported olfactory fluctuations in literature mean the same thing. This problem further emphasizes the need for a standardized questionnaire that provides a clear idea to patients as to what is being asked about this symptom. Given that OF is difficult to quantify and measure, the questionnaire used in this study may prove useful in the future evaluation of OFs in a clinical context.

This study has several limitations. Participants were a consecutive sample of patients that consulted at a tertiary referral center, and the distribution of cases followed the distribution observed in this specific environment. The largest number of consultations were postviral in etiology, the smallest number of consultations were post-traumatic. Although a large sample in total was included, the limited sample size per group precludes very detailed analyses to be done. In addition, patients who experienced olfactory fluctuations not related to persistent olfactory dysfunction were not included in this study and other patient factors (e.g., anxiety, importance of olfaction, environment, etc.) potentially affecting likelihood of reporting presence of OFs were not explored. OFs were reported using self-ratings. Future cohort studies may be done with a larger sample size that includes other patient factors and explores for correlation of OF self-ratings with actual psychophysical measurements of fluctuations and possibly including validation and retesting of the questions used.

## Conclusion

OF is most frequent in sinonasal disease, but was also reported in other causes of olfactory disorders, although at a lesser degree. Self-reported amplitude of OF may help to distinguish postviral disorders from other causes of OD.
